# Barriers to Oral HIV Pre-exposure Prophylaxis (PrEP) Adherence Among Pregnant and Post-partum Women from Cape Town, South Africa

**DOI:** 10.1007/s10461-022-03652-2

**Published:** 2022-03-22

**Authors:** Ivana Beesham, Kathryn Dovel, Nyiko Mashele, Linda-Gail Bekker, Pamina Gorbach, Thomas J. Coates, Landon Myer, Dvora Leah Joseph Davey

**Affiliations:** 1 Desmond Tutu Health Foundation, University of Cape Town, Cape Town, South Africa; 2 Division of Infectious Diseases, David Geffen School of Medicine, University of California Los Angeles, Los Angeles, CA USA; 3 Division of Epidemiology and Biostatistics, School of Public Health and Family Medicine, University of Cape Town, Cape Town, South Africa; 4 Department of Epidemiology, Fielding School of Public Health, University of California Los Angeles, Los Angeles, CA USA; 5 Level One, Wernher Beit North, University of Cape Town (UCT), Faculty of Health Sciences, Anzio Road, Observatory, Cape Town, 7925 South Africa

**Keywords:** Pre-exposure prophylaxis (PrEP), Pregnant, Breastfeeding, South Africa, Adherence, Persistence

## Abstract

Cisgender women, particularly pregnant and postpartum women in Eastern and Southern Africa, face an unacceptably high risk of HIV acquisition. Oral pre-exposure prophylaxis (PrEP) is an effective HIV prevention intervention that can reduce HIV acquisition and vertical transmission. In this qualitative study, we interviewed 21 postpartum women from Cape Town, South Africa who initiated PrEP during pregnancy and who self-reported low PrEP adherence or missed > 1 PrEP follow-up collection. We identified multiple overlapping barriers to PrEP continuation and/or adherence. Individual factors included forgetting to take PrEP daily, being away from home when PrEP should be taken, anticipated stigma and limited disclosure of PrEP use. Women also reported pill-related factors such as side effects and having to take PrEP in addition to other tablets during pregnancy and the postpartum period. Facility-related barriers included logistics around PrEP collection especially when not in antenatal care, as well as transport and financial barriers.

## Introduction

Globally, new HIV infections have decreased substantially since 2010, including a 38% reduction in new infections in Eastern and Southern Africa in the last decade [1]. However, evidence suggests that the risk of HIV acquisition increases considerably during pregnancy and the postpartum period [2]. This may be linked to a combination of biological, social, and behavioural factors that increase susceptibility to HIV [3]. Increased HIV acquisition is not only concerning for women, but for their infants. Cisgender women who acquire HIV during pregnancy or breastfeeding and have not achieved viral suppression on antiretroviral treatment (ART) are at increased risk of transmitting HIV vertically. UNAIDS reported that in 2018, 160,000 children in 23 focus countries were newly infected with HIV—fourfolds higher than the UNAIDS target for that year [4]. One key intervention for the prevention of mother to child transmission (PMTCT) is to prevent HIV acquisition among women of childbearing age [5]. This can be achieved through several strategies including more recently, the use of daily oral pre-exposure prophylaxis (PrEP) to prevent HIV acquisition among high-risk pregnant or breastfeeding women [3]. Oral PrEP is one of the only strategies fully controlled by women themselves, providing autonomy over women HIV prevention efforts. A recent systematic review found that PrEP is safe during pregnancy and breastfeeding [6].

Oral PrEP for at-risk pregnant and breastfeeding women not living with HIV was approved by the South African Department of Health in 2018 [7], but rollout has been slow. There is limited literature on PrEP among this population, but some data suggest that PrEP adherence may be poor, particularly among postpartum women [8, 9]. Daily adherence to oral PrEP is important for achieving adequate levels of protection and studies have found that tenofovir levels are lower during pregnancy and/or postpartum compared to non-pregnant or non-lactating women [10, 11]. While newer long-acting PrEP drugs such as injectable cabotegravir might alleviate some challenges with adherence, it is likely to be several years before these products are widely available for use among pregnant and breastfeeding women. Of concern, a modelling study has predicted that 76,000 new cases of vertical transmission can be expected in South Africa between 2020 and 2030 if PrEP is not taken to scale with high levels of acceptance and persistence [12]. While some studies have identified barriers to PrEP adherence among pregnant and postpartum women such as stigma, overlapping side effects of PrEP use and pregnancy, and remembering to take PrEP [13–16], PrEP services for this population are limited [17], therefore data on PrEP use and adherence to PrEP among pregnant and postpartum women is sparse.

In this qualitative analysis, we explore barriers to PrEP adherence and continuation among pregnant and postpartum women from Cape Town, South Africa who started oral PrEP during pregnancy and self-reported low adherence to PrEP or completely discontinued PrEP use. Understanding the factors that affect adherence to daily oral PrEP among this population is critical for epidemic control given that pregnant and breastfeeding women in South Africa are at high-risk of HIV acquisition and vertical transmission.

## Methods

We nested a qualitative study within the larger PrEP in pregnancy and postpartum (PrEP-PP) study (NCT03902418). The PrEP-PP study is a prospective cohort study being conducted at a single primary health care, public clinic in Cape Town, South Africa to assess PrEP initiation, continuation and adherence in a cohort of pregnant and breastfeeding women. PrEP-PP began enrolling pregnant and breastfeeding women in August 2019 and follow-up was ongoing as of March, 2022. Women who attend antenatal care and are HIV-negative, pregnant, aged ≥ 16 years, and have no medical contraindications to PrEP are eligible for the parent study. Women enrolled in the study are counselled on the risks of HIV acquisition during pregnancy and postpartum and offered oral daily PrEP from pregnancy until 12-months postpartum. Women can start or stop PrEP at any time in the study. PrEP is integrated into antenatal care and offered from the first antenatal visit onward. Women who agree to initiate PrEP are given a monthly supply of Truvada® (tenofovir disoproxil fumarate/emtricitabine). Study visits are conducted at 1-month after PrEP initiation, then 2-months after, then every 3-months up to 12-months post-partum. PrEP providers are trained staff including a trained nurse practitioner, PrEP counsellors and study interviewers.

For this qualitative sub-study, we purposively recruited women from the PrEP-PP cohort to participate in a one-time in-depth interview. We invited women who reported poor PrEP adherence (either missed a PrEP refill or used PrEP intermittently [n = 13, 62%]) or discontinued PrEP during pregnancy or postpartum (n = 8, 38%). Eligibility criteria for the sub-study included being enrolled in PrEP-PP for a minimum of 6 months, being postpartum with a live birth at the time of recruitment for the sub-study, and self-reported low adherence to PrEP during pregnancy and/or postpartum (defined as missing ≥ 6 doses of PrEP within the last 30 days), and/or missing > 1 follow-up PrEP distribution appointment (missed ≥ 14 days visit measured through study file review). Study staff invited women to participate in in-depth interviews between August and October 2020. Study staff reviewed study files to identify individuals who were eligible. These individuals were recruited via phone and in person during routine facility visits until we reached thematic saturation during data analysis (until no new themes emerged in five or more interviews) [18].

The interview guide explored reasons for study participation and initiating PrEP, experiences with PrEP, including disclosure of PrEP use, and factors that influenced adherence to PrEP. The interview guide was developed, and pre-tested prior to the sub-study. Interviews were conducted in-person when participants were already in the facility for study follow-up or by telephone due to the COVID-19 lockdown and associated restrictions. Where interviews were conducted in-person, a private room at the facility was used to ensure confidentiality. For telephone interviews, participants were asked to sit in a private space to ensure confidentiality. Interviews lasted approximately 30–40 min. Interviews were conducted in isiXhosa (local language) by a single female, native isiXhosa speaking interviewer (NT). The interviewer has extensive experience conducting qualitative interviews, was not part of the PrEP-PP study, and had no contact with the study participants prior to the interview.

All interviews were audio-recorded, transcribed, and translated into English. Field notes were also recorded. Interviews were transcribed by a trained research staff (YM). All transcriptions were then reviewed by the interviewer for accuracy and necessary corrections were made.

We used a thematic approach for coding and analysis of the interviews [19]. Interviews were coded and analysed using Nvivo 12 (QSR International, Victoria, Australia) [20]. A code list was developed by three trained research assistants (YG, JMM, NT) and led by the study principal investigator (DJD). The coding (within transcripts) was reviewed and revised by IB. Themes were developed a priori based on existing literature on HIV adherence and continuation during pregnancy and inductively during the process of coding. The codebook was also guided from quantitative data (self-reported barriers to PrEP use) from baseline and quarterly participant surveys from the PrEP-PP parent study. The analysis integrated memo writing and concept mapping to refine understandings of PrEP adherence and develop themes that were associated with low adherence to PrEP [18]. We selected quotes from women and present with their participant identification (PID) and maternal age. We used The Consolidated Criteria for Reporting Qualitative Research (COREQ) checklist for analysis and reporting [21].

We adapted Ickovics’ and Meisler’s conceptual framework for the analysis that evaluates factors affecting treatment adherence in HIV clinical trials [22] to assess factors associated with poor continuation and low adherence to PrEP in pregnant and postpartum women. The framework posits that adherence is affected by factors across five levels including: (1) Individual (internal motivation; biofeedback), (2) Treatment regimen (daily oral PrEP), (3) Patient-provider relationship, (4) Facility setting, and (5) Disease (HIV and HIV risk in self and partner). In our analysis, we considered the following factors for PrEP adherence and continuation: (1) individual-level factors (e.g., relationship status and communication, disclosure of PrEP use and anticipated or experienced stigma); (2) pill-related factors (e.g., side effects, pill burden); (3) clinical setting/facility-level factors (e.g., access to PrEP, transport and financial barriers to care).

Ethical approval was obtained from the University of Cape Town (#297/2018) and the UCLA Institutional Review Board (IRB#18-001622). Written informed consent was obtained from all women to participate in the interviews. Women who were interviewed were reimbursed R120 (approximately $8 USD) in grocery vouchers for their time and transport costs.

## Results

### Participant Characteristics

We contacted 26 postpartum women who had initiated PrEP during pregnancy but reported sub-optimal adherence to PrEP or discontinued using PrEP, of which, 21 women agreed to do the in-depth interview (81% acceptance). The mean age was 29 years, two-thirds (67%) had secondary education or higher, fewer than half (43%) were married or cohabiting with their partner, and most women (81%) reported that their partner was HIV-uninfected (Table 1). Women were a mean 33 weeks postpartum (8.3 months) at the time of the interview.

**Table 1:** Participant characteristics at enrolment into the PrEP-PP study (n = 21 postpartum women on PrEP)

Characteristic	n (%) or mean
Age at study enrolment (years)	29
Gestational age at study enrolment (weeks)	22
Employed or full-time student	15 (71)
Partner’s HIV status	
Negative	17 (81)
Unknown	4 (19)
Education—secondary level or higher	14 (67)
Married or cohabiting with partner	9 (43)
Has ≥ 1 child before index pregnancy	12 (57)

While we describe findings by level in the Ickovics’ and Meisler’s conceptual framework, barriers were often overlapping and cumulative, with multiple individual-barriers interacting with pill- or facility-level barriers to ultimately deter PrEP continuation and/or adherence.

### Individual-Level Factors

Women reported several individual-level factors that acted as barriers to daily PrEP adherence and continuation. The most common barriers included forgetting to take PrEP, and travel or being away from home when PrEP needed to be taken. In addition, women expressed that they were fearful about anticipated stigma from family and friends who may think that they were HIV-infected and taking ART, which limited their disclosure of PrEP use.

#### Forgetful to Take PrEP Daily

Women reported that they frequently forgot to take their daily PrEP. This was often intermittent, as one woman reported, “I was taking it and forgetting it on some other days” (PID200, 20 years), or in some cases, missed doses primarily occurred soon after PrEP initiation but were resolved over time, “When I started taking PrEP, I would miss it, but it was not always. I would miss it, but they [husband and children] would remind me. I didn’t always remember it.” (PID395, 43 years).

Some women tried to ensure that they remembered to take PrEP for the first 7 days to achieve adequate levels of protection before having condomless sex, despite being forgetful after when not planning to have condomless sex, “I ensure that I take it, but I don’t want to lie, there are times when I forget. I forget it at times but I make sure about the 7 days.” (PID217, 26 years).

#### Being Away from Home When PrEP Needed to be Taken

Some women reported not taking PrEP daily as prescribed because they were away from home and they did not take PrEP with them. One woman reported, “Sometimes I am not home when the time comes [to take PrEP]. I often forget to put my pills in my bag.” (PID220, 30 years). Similarly, another participant reported that when visiting her mother, she would forget to take her PrEP pills, “When I started taking PrEP, I was living with my father, now I am living with my mother. So, when I was living with my father and I would visit my mother, I would forget to take the pills with and that is how I would forget.” (PID217, 26 years). In some instances, unplanned trips away from home resulted in women missing extended PrEP doses. One woman reported that the unexpected hospitalization of her child resulted in her not taking her PrEP for about 2 weeks:

Yes, because I left home unexpectedly [child was hospitalized], and I didn’t have it [PrEP tablets] with me. When I was there [in hospital], I didn’t even think about it [PrEP] at all. I only had it a day before my baby was admitted. I stayed for ten if not fourteen days. I didn’t take it [PrEP] at all, while I was there. (PID200, 20 years)

Travel, moving, and family illness were also barriers to consistent PrEP use in pregnant and postpartum women on PrEP.

#### Stigma

Several women reported anticipating stigma relating to PrEP use from partners, family members or the community. In most cases, women reported that they feared that people would think that they were HIV-infected and taking ART. “I was worried because I thought they [close contacts] were going to judge me or think that I am HIV positive.” (PID262, 27 years). Another woman reported, “It seems like they think it [PrEP] is for HIV, that I am taking a pill for HIV because they don’t know, they have never heard about it [PrEP] before.” (PID 217, 26 years). In some cases, anticipated or experienced stigma influenced PrEP initiation, adherence and continuation. One woman reported that she did not initiate PrEP immediately upon receiving it due to negative responses she had heard from people who lived with her and her community, “I did not start taking my tablets straight after my first visit … I think I listened to people, and even here at home they were saying negative things about PrEP. They were associating it with HIV …. I was afraid to take it.” (PID309, 28 years). Additional concerns from women included the association of using PrEP with being promiscuous. “I was thinking that they [partner, friends, family members] will say I sleep around …. at the beginning I was in and out of PrEP.” (PID200, 30 years).

Other women were afraid of partner reactions when she disclosed, or he inadvertently found out about her PrEP use. Women reported multiple concerns regarding their partners’ response. Some anticipated that partners may assume she has HIV (assuming PrEP is ART), if not already disclosed her PrEP use, the partner may feel that she was trying to hide taking PrEP and become upset, question the level of trust and stability in the relationship (i.e., why does she need PrEP?), and/or raise concern about PrEP safety and potential harm for the baby. One woman reported, “I was concerned but I thought that I should disclose it and not hide it because how would it look like when he sees me taking something he doesn’t know, maybe it would upset him” (PID 024, 35 years). Another woman reported that although she was concerned about disclosing PrEP use to her partner, he did not object to her taking PrEP, “My concern when I came back with it [PrEP] …. I was concerned about telling the partner about PrEP. I thought he was going to tell me that I don’t trust them. But when I told him, he didn’t have a problem” (PID220, 30 years). For some women, anticipated stigma was associated with PrEP discontinuation:

People contributed to my decision to stop, because I would have had to explain to people such as my boyfriend when they find out that I am taking PrEP .... Most people were not going to buy the story that I tell them about how PrEP works.… its things like that that made me to stop, I thought what other people were going to think about me when they hear that I am taking PrEP. (PID405, 23 years).

#### Disclosure-Related Factors

For most women, disclosure was not reported as a barrier to PrEP use or continuation. All women reported that they had disclosed their HIV status and PrEP use to at least one individual, usually to their partner, and many women reported disclosing to partners, family members (including parents, siblings and children) and friends. Reasons for disclosing PrEP use included trusting the individual and not wanting to keep secrets, receiving support from the individual such as reminders to take PrEP and emotional support, concern that if she did not disclose others would assume she was HIV-positive (and PrEP was ART), and perceiving others as at risk for HIV and suggesting that they should also consider taking PrEP. One woman who had disclosed to her mother, partner and friend said, “They are people I trust, and they are people who won’t judge me about why I am taking this thing. I thought they won’t judge me, and they will support the decision I make.” (PID217, 26 years). Another woman felt she should not keep secrets from her partner since he will likely find the pills anyway, “I don’t like having secrets. I thought that if I hide these pills, one day he [partner] will ask me about them. One day he will find them, ask me what pill I am taking. I thought I should explain that there is this pill.” (PID220, 30 years).

Despite concerns regarding anticipated stigma, very few women reported actually experiencing negative responses upon disclosing PrEP use. Most said individuals were neutral or supportive regarding their PrEP use. A woman who disclosed to her aunt and partner said, “They never had any issues, they just said I made a good decision after I explained to them.” (PID262, 27 years).

Few women reported mixed or negative responses to PrEP use following disclosure. One woman said, “He asked why I cared about the pills [PrEP] and that we should end our relationship. I thought he was crazy, he was speaking nonsense.” (PID295, 21 years). Another woman reported mixed reactions after disclosing PrEP use to several individuals:

My mother was surprised, she asked a lot of things. She asked what was happening, was I not trusting the father of my child. Did he had sex with someone else? I said no, I know he has his side affairs. My partner was furious but I told him that he knows how he behaves and that he doesn’t give me any choice but to protect myself. My friends they are very supportive to an extent that they also wanted to enrol on PrEP but they do not know how to get it since they are not pregnant. (PID200, 20 years)

Yet most women reported that such negative responses did not affect their PrEP continuation, “I had doubts when my friend told me that I am just like someone who takes pills for HIV because I will take them for life. But I realised that this is my life.” (PID334, 29 years). Postpartum women were able to disclose their PrEP use to their family and community which contributed towards improved PrEP use.

### Reduced Sexual Activity or Abstinence During Pregnancy and/or Postpartum and PrEP Use

Many women reported reduced sexual activity or abstinence for periods during the pregnancy and/or postpartum, however this was not reported as a reason for discontinued or intermittent PrEP use. Several women continued using PrEP even while abstaining from sex for months, for example, one woman reported she stopped having sex from the 8th month of her pregnancy until 3 months postpartum, but she continued using PrEP: [{\text{Al}}({\text{OH}})_{x} ]_{{{\text{gel}}}}^{{n - }} \to {\text{Al(OH}})_{{\text{3}}} \downarrow {\text{ + }}\left( {x - {\text{3}}} \right){\text{OH}}^{ - }

Interviewer: So, you continued taking your PrEP when you weren’t having sex?
Participant: Yes, I was taking it. (PID 024, 35 years old).

### Pill-Related Factors

#### Anticipated or Experienced Side Effects

Many women reported missing doses of PrEP or discontinuing PrEP altogether due to experiencing PrEP side effects such as vomiting, nausea and dizziness. One woman reported early side effects affecting adherence, “Yes, I have missed it when I was new on it, it used to make me feel nauseas or feel dizzy sometime” (PID 264, 21 years), but the side effects later resolved, and adherence improved. Other women said they discontinued PrEP due to side effects, “…. they [PrEP pills] were making me vomit …. so I decided to stop them” (PID199, 28 years).

Some women missed doses of PrEP due to anticipated side effects, rather than having actually experienced any side effects, “There were times when I didn’t take the pill because I thought that I will get a headache and be nauseous if I take it.” (PID217, 26 years).

#### Other Pill-Related Factors

A few women reported other factors related to taking pills that influenced their adherence such as a dislike for taking tablets, difficulties with committing to daily pill taking and challenges taking PrEP in addition to other tablets. Several women in our study were also taking tablets for pregnancy, post-delivery or for other illnesses. These tablets included vitamins for pregnancy and analgesics post-delivery. One woman reported challenges associated with taking multiple medications:

I don’t like pills and I was also taking pills for the pregnancy. I thought these ones [PrEP] should wait because I was taking pills for the pregnancy…. I gave birth through a c-section [caesarean], so I stopped [PrEP]. I was taking pills for the operation, so I felt that they were too many. I put PrEP aside and focused on my baby and took the other pills, for the operation. (PID334, 29 years)

Another woman reported challenges with overlapping side effects when taking PrEP along with medication for hypertension and arthritis, and difficulties ascertaining which tablets were causing side effects:

Yes, I have missed it [PrEP] when I was new on it, it used to make me feel nauseas or feel dizzy sometime. However, I told the nurse but she was not sure which tablets exactly were causing side effects between the arthritis, hypertension and PrEP tablets I was taking … (PID 264, 21 years)

### Clinical Setting/Facility-Related Factors

Some women reported that access and facility-related factors affected PrEP adherence and continuation, particularly attending follow-up PrEP visits.

#### COVID-19 Related Factors

Some women reported that they were unable to attend clinic visits and collect PrEP due to the COVID-19 pandemic and related lockdowns. One woman reported, “So, because of transport issues and the lockdown I missed a visit date [and PrEP refill].” (PID295, 21 years). Another woman reported that access to healthcare facilities was related to the different lockdown levels. “However now COVID-19 hinders us from accessing health facilities. Especially during the time when we had very high cases but its better now that we are on level two [lockdown level with less restrictions] ….” (PID185, 43 years). Fear of COVID-19 exposure and lockdown presented barriers to attending the clinic visits and receiving repeat prescriptions for PrEP.

#### Limited Access Postpartum

Increased barriers to postpartum PrEP access were reported by some women, which included transport, logistical, financial and access related barriers to consistent clinic attendance. Since postpartum women no longer attended the facility for antenatal care, they had to make special facility visits just to receive PrEP, dramatically increasing the burden of PrEP use since facility visits included transportation cost and extended wait times. One woman reported that upon returning from the rural area, she did not have money to come to the clinic. “I came back from the rural area and didn’t have money to come to the clinic. I came late …. When I came back, I couldn’t come here [to the clinic for PrEP], because my husband wasn’t working.” (PID106, 24 years). Another woman reported missing her clinic visit because in addition to not having money to access transport, she also had no one to look after her baby.

One woman reported that she was unable to access PrEP when she changed clinics after the delivery of her child to attend a postnatal clinic, as the clinic only provided services to children:

I do not think they have it [PrEP] there [postnatal clinic], because when we get there we go to the nurses to get the injections [vaccination] for our kids and we only speak about baby related matters and so there is completely nothing that has to do with us as mothers … I haven’t reconnected with PrEP services because my baby and myself are no longer attending here in this clinic, I take my baby to a different facility for postnatal care and there hasn’t been any conversation about the PrEP service so I won’t know whether they offer it or not. (PID199, 28 years)

## Discussion

Our study findings provide insights into factors affecting oral PrEP use among postpartum women who self-reported low adherence to PrEP or who missed study visits. We found that adherence to PrEP was negatively influenced by multiple overlapping barriers. Individual factors such as forgetting to take daily PrEP and being away from home when PrEP needed to be taken contributed to some women missing doses of PrEP. Several women in our study anticipated stigma related to PrEP use because they were afraid taking PrEP would be associated with having HIV (if PrEP was mistaken as ART), or that people would assume PrEP was being used because they were promiscuous. Fear of stigma led to some women discontinuing PrEP. Pill-related factors such as side effects, anticipated side effects and having to take PrEP in addition to other pills for pregnancy or post-delivery resulted in some women missing doses of PrEP or discontinuing altogether. Lastly, we found that facility-related factors were barriers to PrEP use among some women. Transport and financial barriers to accessing care, the lack of availability of PrEP at some postpartum clinics, and the COVID-19 pandemic and related lockdowns impacted PrEP adherence and continuation, especially for postpartum women who did not regularly attend antenatal clinics. Given that PrEP research for pregnant and postpartum women is limited [17], our study findings improve understandings of barriers to PrEP adherence and continuation and can be utilized by PrEP delivery programmes for pregnant and postpartum women.

Other studies have identified similar barriers to PrEP adherence among pregnant or breastfeeding women in other settings. Qualitative studies have identified remembering to take PrEP, side effects, and perceived HIV stigma as potential barriers to PrEP use among this population [13, 14]. In a study among HIV-uninfected pregnant women aged 18–24 years, from rural South Africa, more than half the women felt that taking PrEP would be associated with having HIV [14]. A study that looked at actual PrEP use among pregnant, HIV-uninfected women from Kenya in serodiscordant relationships found there were overlapping side effects from pregnancy and early PrEP use, such as nausea, dizziness, fatigue and gastrointestinal alterations, all of which acted as a barrier to PrEP continuation and adherence during pregnancy [15]. A large PrEP implementation project conducted in Kenya among pregnant and postpartum women found that less than 40% of women continued PrEP at month 1, and frequent reasons for PrEP discontinuation were side effects and perceived low HIV risk [8].

Like our findings, the HPTN082 study found that stigma had a negative influence on PrEP adherence and continuation among non-pregnant women [23]. Increased awareness about PrEP not just among pregnant women, but also the larger communities in which these women live is essential to promote PrEP use and remove suspicions and lack of knowledge about PrEP. This should be a combined effort from health and other sectors such as social services and networks.

In our study, we found that all women had disclosed PrEP use, and most women disclosed to their partners. Disclosure of PrEP use has varied in recent studies, for example in HPTN082, only 40% of women planned to disclose PrEP use [24], while in the ECHO trial, at one research site, 90% of women had disclosed PrEP use [25]. Reasons for high rates of disclosure among our study participants might be because women in our study were older, pregnant, and reported close, supportive relationships with family members and partners, which might be different to other populations such as adolescent girls and young women. In addition, close to half of the women in our study were married or cohabiting with their partners.

We identified important logistical, transport, financial and access-related barriers that affected PrEP adherence. These are important to address as we consider the wider rollout of PrEP among pregnant and postpartum women in South Africa. In addition, we need to consider the context and structure of local health care services in the country as maternal child health services are often disaggregated, more so in urban areas. Furthermore, postpartum services in general may be lacking in some areas. While some barriers such as the effects of the COVID-19 pandemic and related-lockdowns might have been unavoidable, we should attempt to find innovative ways to address other barriers, including the use of implementation science to guide PrEP delivery [26] and requiring less frequent PrEP visits to minimize logistical barriers to care.

An additional important consideration is the timing of barriers in the PrEP cascade. Barriers such as forgetting to take PrEP and PrEP related side effects tended to occur in the initial weeks following PrEP initiation, whilst difficulties in accessing PrEP refills were frequently in the post-partum period when women attended different health care facilities or travelled to their family home (in distant locations) post-delivery. Other barriers such as stigma were reported around PrEP initiation and continued use. Strategies to improve adherence and continued use should take into consideration when barriers are likely to be experienced during the PrEP cascade. Side effect counselling and management are important at PrEP initiation and in the subsequent weeks. Differentiated models for PrEP delivery such as pharmacy delivery, community based or mobile clinics, and integration of PrEP into family planning and well-baby services to ensure PrEP counselling and drug access in postpartum. PrEP counselling sessions should include discussions about travel and movement and where possible, additional supplies of PrEP, or referral to other facilities should be provided. Finally, longer acting PrEP drugs such as injectables, effective at preventing HIV in cisgender women [27], and implants might address barriers to daily pill taking and are more discrete to allow women to use a method without the knowledge or consent of their partner.

Recent research has focused increasingly on the “effective use of PrEP” which considers that PrEP users might have periods/episodes of risk where PrEP is needed but can safely discontinue PrEP when it is not required [28]. Alternatively, other users might have frequent, indefinite risk to HIV and continuous, uninterrupted PrEP use is necessary [28]. In our study, we found that many women continued using PrEP during periods of sexual abstinence during pregnancy and/or the postpartum period. Education and counselling on importance of daily oral PrEP use prior to, and during periods of sexual activity for both PrEP providers and users may improve adherence.

In addition to some pregnant and postpartum women abstaining from sex during pregnancy and/or the postpartum period, there are other factors affecting PrEP adherence that may be unique to pregnant and/or postpartum women compared to other populations, for example, overlapping side effects of PrEP drugs and early pregnancy symptoms, having to transition from antenatal to postnatal healthcare facilities and then to general clinics which may be located at different sites, being hospitalized for delivery, and travelling home post-delivery for weeks to months to locations that might not have PrEP services. However, PrEP can be integrated into ante and postnatal care as previous studies have shown [8, 9]. PMTCT services were successfully integrated into antenatal services nationally in South Africa and are now widely available. It is therefore possible that over time, PrEP services can be successfully integrated and scaled up for pregnant and postpartum women.

Our study has some limitations. Women were interviewed from one urban study site in Cape Town, South Africa, and therefore may not be representative of other geographical regions or populations. Furthermore, interviews were conducted during the postpartum period, therefore there might be recall bias. Women in our study were older, and all women in our study had disclosed PrEP use, and most had disclosed to their partners, therefore our findings might not be generalizable to other populations. Lastly, adherence to PrEP might have fluctuated over time, for example, women who missed PrEP doses initially due to side effects, might have taken PrEP more regularly once side effects subsided.

## Conclusions

Given that data on PrEP use among pregnant and postpartum women are limited, and that the risk of HIV increases considerably during pregnancy and the postpartum period, our study provides important insights on reasons pregnant and postpartum women have poor adherence or discontinue PrEP. Barriers to PrEP use include individual factors such as forgetting to take PrEP and anticipated stigma; pill-related factors such as side effects and having to take PrEP in addition to other tablets; and facility-related factors such as logistical, financial and transport barriers to attending clinic visits, particularly postpartum when women do not frequent facilities. Counselling, effective management of side effects, and restructuring PrEP delivery to reduce logistical barriers to care postpartum, including integrating PrEP into well-baby visits and community models, are urgently needed as PrEP is rolled out to pregnant and postpartum throughout South Africa and in the region.

## Data Availability

Data available upon request to dvoradavey@ucla.edu.

## Code Availability

Code available upon request to dvoradavey@ucla.edu.
