# Correction to: Barriers to Oral HIV Pre-exposure Prophylaxis (PrEP) Adherence Among Pregnant and Post-partum Women from Cape Town, South Africa

**DOI:** 10.1007/s10461-022-03676-8

**Published:** 2022-04-21

**Authors:** Ivana Beesham, Kathryn Dovel, Nyiko Mashele, Linda-Gail Bekker, Pamina Gorbach, Thomas J. Coates, Landon Myer, Dvora Leah Joseph Davey

**Affiliations:** 1 Desmond Tutu Health Foundation, Level One, Wernher Beit North, University of Cape Town (UCT), Faculty of Health Sciences, Anzio Road, Observatory, Cape Town, 7925 South Africa; 2 Division of Infectious Diseases, David Geffen School of Medicine, University of California Los Angeles, Los Angeles, CA USA; 3 Division of Epidemiology and Biostatistics, School of Public Health and Family Medicine, University of Cape Town, Cape Town, South Africa; 4 Department of Epidemiology, Fielding School of Public Health, University of California Los Angeles, Los Angeles, CA USA

## Correction to: AIDS and Behavior 10.1007/s10461-022-03652-2

In the original version of this article, An erroneous following text was included in the “Reduced Sexual Activity or Abstinence During Pregnancy and/or Postpartum and PrEP Use” section and this has been removed

Erratum text:

$$[{\text{Al}}({\text{OH}})_{x} ]_{{{\text{gel}}}}^{{n - }} \to {\text{Al(OH}})_{{\text{3}}} \downarrow {\text{ + }}\left( {x - {\text{3}}} \right){\text{OH}}^{ - } $$

